# Molecular Research and Treatment of Breast Cancer 2.0

**DOI:** 10.3390/ijms25073932

**Published:** 2024-04-01

**Authors:** Anna Kawiak

**Affiliations:** Intercollegiate Faculty of Biotechnology, University of Gdansk, Abrahama 58, 80-307 Gdansk, Poland; anna.kawiak@biotech.ug.edu.pl

Breast cancer is the primary contributor to cancer-related deaths among women. The intricacy of the molecular mechanisms underlying the onset and advancement of tumors contributes to the varied nature of breast cancer. This molecular diversity presents a significant obstacle in selecting treatment options and predicting disease outcomes. Progress in molecular research has led to deeper insights into the cellular pathways determining breast tumor progression. Such advancements have paved the way for recognizing novel diagnostic markers and developing innovative therapeutic approaches, a selection of which is presented in this Special Issue.

Breast cancer molecular subtypes are distinguished based on the histopathological assessment of primary tumors, the expression levels of hormone receptors (estrogen receptors (ERs) and progesterone receptors (PRs)), and epidermal growth factor receptor 2 (HER2/Neu), alongside additional genomic and transcriptomic profiling. These subtypes include normal breast-like, luminal A (ER+/PR+ with low Ki67), luminal B (ER+/PR+ with HER2+ or HER2− and high Ki67), HER2-enriched (HER2+), basal-like, and claudin-low. Apart from the diversity of these molecular subtypes, patient-specific intertumoral variations, as well as intratumoral heterogeneity in the cell populations within a patient’s primary tumor and metastases, can significantly impact their treatment choices and outcomes [1].

Luminal, hormone-receptor-positive breast cancers comprise around two thirds of breast cancer cases [2]. The predominant therapeutic approach to hormone-receptive breast cancers is targeting their ERs or suppressing estrogen production. Endocrine therapies include selective estrogen receptor mediators (SERMs), selective estrogen receptor degraders (SERDs), and aromatase inhibitors (AIs). Despite the clinical advantages of endocrine therapy, around 20% of patients develop de novo or acquired resistance, which poses a significant clinical hurdle for individuals with hormone-responsive breast cancer. The mechanisms underlying endocrine resistance are complex, with a loss of ER expression occurring in only around 10% of endocrine-resistant breast tumors. More frequently, endocrine resistance is driven by the hormone-independent activation of ERs, which either involves gain-of-function mutations in the ER, changes in ER and coactivator/corepressor interactions, or the compensatory cross-talk between ERs and growth factor receptor/oncogenic signaling pathways [3]. Endocrine resistance can also occur due to alternative splicing, which contributes to the formation of alternative splice variants. In the case of tamoxifen, a SERM used in the first-line treatment of ER+ breast cancer, a novel variant BQ323636.1 (BQ) of the nuclear receptor corepressor 2 (*NCOR2*) has been shown to mediate tamoxifen resistance [4]. Research has shown that tamoxifen participates in increasing BQ’s expression through the induction of DNA damage and the activation of ATM/CHK2 signaling. CHK2 induces the phosphorylation of BQ, increasing its stability. In vivo analysis showed that the high nuclear expression of p-CHK2 correlated with nuclear BQ expression and conferred tumor resistance to tamoxifen. Targeting CHK2 reduced this phosphorylation and enhanced the polyubiquitination of BQ, reducing its expression and reversing its tamoxifen resistance. A high p-CHK2 expression in clinical tissue samples correlated with poorer overall survival, indicating the clinical significance of CHK2 and its targeting in overcoming tamoxifen resistance [5].

Identifying the targets involved in the endocrine therapy cross-resistance of both SERMs/SERDs and AIs can offer valuable insight into increasing the efficacy of endocrine therapy. A recent study focused on the analysis of data obtained from the clinical TCGA-BRCA PanCancer Atlas database, identifying gene candidates involved in SERMs/SERDs and AI cross-resistance. Transcriptomic and proteomic analysis revealed a set of candidate targets involved in both SERMs/SERDs- and AI-mediated resistance. These candidate gene sets effectively distinguished between progress/resistant groups (PD) and complete response groups (CR), and they demonstrated significant correlations with the survival outcomes of both groups, providing novel insights into the mechanisms and therapeutic strategies involved in improving endocrine therapy outcomes in the future [6].

Apart from genetic alterations, epigenetic modifications, including DNA methylation, RNA modifications, and histone modifications, play an important role in breast cancer oncogenesis. One of the most common mRNA modifications in eukaryotic cells includes N6-methyladenosine (m6A) alterations, which impact RNA transcription, processing, splicing, degradation, and translation [7]. The regulators of m6A are ‘writers’ (methyltransferases), ‘erasers’ (demethylases), and ‘readers’ that regulate m6A modifications [8]. The m6A reader YTH N^6^-methyladenosine RNA binding protein 1 (YTHDF1) has been implicated in the initiation and development of breast cancer. The research of Luo et al. (2024) examined the association between the expression of YTHDF1 and the clinicopathology of breast cancer. YTHDF1 was found to be overexpressed in breast cancer tissue and was associated with a poor prognosis. YTHDF1 was identified as a crucial element of the m6A regulatory proteins in breast cancer and is thought to play a role in the immunological microenvironment. Furthermore, miR-378g was identified as an inhibitor of YTHDF1 expression and breast cancer cell proliferation, indicating the potential of YTHDF1 as a target for breast cancer therapy [9].

The deregulation of steroid hormone receptors is associated with breast cancer oncogenesis. In addition to the estrogen receptor (ER) and progesterone receptor (PR), the androgen receptor (AR), which is overexpressed in around 70–90% of breast tumor occurrences, has also been implicated in the disease’s initiation and development. Interestingly, the role of the AR differs among various breast cancer subtypes. In ER+ breast cancer, the AR has tumor-suppressing activity, interfering with the functioning of the ER, whereas, in ER- breast cancer, the AR has demonstrated oncogenic activity, inducing the activation of the signaling pathways involved in tumor progression. Studies have shown the efficacy of androgens in reducing the risk of hormone-induced breast cancer. A phase II clinical trial evaluating a selective AR-activating agent, enobosarm, has suggested it may have clinical benefits in ER+ metastatic breast cancer patients. Reports from clinical studies have demonstrated that androgen inhibitory agents, alone or in combination, have demonstrated their clinical benefits in ER−/AR+/HER2+, AR+/ER−/PR−, and AR+/TNBC patients [10].

Triple-negative breast cancer (TNBC) accounts for up to 20% of breast cancer cases and is characterized by the absence of ER, PR, and HER2 expression. In gene expression profiling, TNBC is frequently characterized as a subtype of basal-like breast cancer, tends to have a more aggressive phenotype compared to other breast cancer subtypes, and is associated with poorer overall prognosis [11]. The treatment of TNBC mainly involves chemotherapy. However, with the advancement of immunotherapy and the introduction of PD1/PDL1 checkpoint inhibitors, the treatment outcomes of TNBC have improved. A promising therapeutic approach in TNBC-directed immunotherapy includes targeting the natural killer group 2D ligand (NKG2DL) expressed in TNBCs. NKG2DLs trigger antitumor immune responses by activating natural killer (NK) cells and T cells upon binding to the NKG2D receptor. Various therapeutic strategies targeting the NKG2DL/NKG2D axis are currently being developed. Recently, bispecific fusion proteins (BFPs) comprising an NKG2D receptor domain targeting multiple NKG2DLs fused to anti-CD3 (NKG2D-CD3) or anti-CD16 (NKG2D-CD16) Fab fragments have been developed. The NKG2D-CD3/CD16 BFPs were found to activate and direct NK and T cells to TNBC cells, resulting in their elimination, pointing to NKG2D-mediated NK and T cell engagers as a potential option for the treatment of TNBC [12].

A subset of TNBCs cluster into a molecular subtype, named claudin-low, which is negative for ER, PR, and HER2 expression and displays mesenchymal cell features. Claudin-low tumors have a reduced expression of epithelial tight junction and cell-to-cell adhesion proteins, such as claudin 3, 4, 7 (CLDN 3, 4, 7), and E-cadherin. These tumors express markers associated with an epithelial–mesenchymal transition (EMT) and exhibit mammary stem cell features. Claudin-low breast cancer is associated with increased invasiveness and worse patient prognosis [13]. The search for therapeutic targets for claudin-low breast cancer is still ongoing. Recent studies have revealed an association between this aggressive breast tumor phenotype and the expression of cytochrome P4501B1 (CYP1B1). In cells with a claudin-low phenotype, the knockdown of CYP1B1 resulted in the loss of their mesenchymal phenotype, increased their claudin expression levels, decreased their migration and invasive capacity, and increased their chemosensitivity. An analysis of clinical breast tumor samples revealed a correlation between CYP1B1 expression, reduced CLDN7 levels, and poor prognosis. These findings point to the potential of CYP1B1 inhibitors as a strategy for adjuvant or neoadjuvant therapy in metastatic breast cancer [14]. Another factor studied in the context of breast cancer metastasis, presented in this Special Issue, is Irisin (Ir), an adipocytokine involved in the regulation of metabolic processes, formed from fibronectin type III domain-containing protein 5 (FNDC5). Research on the implications of FNDC5/Ir expression in oncogenesis has revealed its association with prognostic significance and its potential inhibitory effects on metastasis. In the context of breast cancer, FNDC5/Ir expression was found to be increased in tumor tissues and was a good prognostic factor. Moreover, higher FNDC5/Ir levels were determined in breast cancer patients without lymph node metastasis, indicating a potential role of FNDC5/Ir in the inhibition of EMT [15]. Further analysis showed a positive correlation between FNDC5/Ir and E-cadherin expression. However, weak to moderate correlations were observed between FNDC5/Ir and EMT-inducing transcription factors, indicating that their interactions are complex. Serum Ir levels were associated with lymph node metastasis and histological grades but did not reflect tumor tissue levels, implying that further elucidation of the mechanism of Ir’s release into the plasma is required [16].

Delineating the correlation between deregulations in the functioning of organelles and breast cancer pathogenesis has revealed potential treatment strategies. Aberrations in the processes associated with the Golgi apparatus, such as protein glycosylation, vesicle transport, and interactions between the Golgi apparatus and microtubules, have been linked with the initiation and progression of breast cancer [17]. Several Golgi-associated proteins have been connected with Golgi-mediated breast cancer oncogenesis. GOLPH3 (Golgi phosphoprotein 3) has been identified as an oncoprotein and is overexpressed in breast cancer, correlating with poor prognosis. The role of GOLPH3 in oncogenesis is associated with the altered regulation of several cellular processes, including the control of Golgi-to-plasma membrane transport, promoting the secretion of factors that support the cancerous phenotype; the glycosylation enhancement of cancer-related proteins; the endocytosis regulation of signaling pathway effectors; and Golgi structure reorganization upon DNA damage [17,18]. Rab GTPases, a family of small GTPases that regulate the intracellular membrane trafficking pathways, impact various cellular processes associated with breast cancer, including invasion and metastasis. For example, in ER+ breast cancer, Rab27b activates MMP2 secretion, promoting cell invasion. Another RAB GTPase, RAb2a, regulates the post-endocytic transport of MT1-MMP, a metalloprotease crucial for extracellular matrix restructuring. Furthermore, Rab2a controls the transport of E-cadherin in the Golgi apparatus, influencing the cell–cell adherence junction and breast tumor cell invasions [17,19]. Understanding the intricate functions of the various proteins participating in Golgi functioning can provide valuable insight into the development of targeted therapies for breast cancer management. One such candidate, targeting the Golgi Membrane Protein 1 (GOLM1), which is involved in the metastasis of cancer cells, is the polyphenol epigallocatechin-3-gallate (EGCG). EGCG has been shown to inhibit the activity of matrix metalloproteinase 2 and 9 in multidrug-resistant human breast cancer cells, demonstrating its anti-invasive properties. In addition to its anti-invasive and anti-migratory properties, EGCG exerts diverse effects on breast cancer cells, inhibiting oncogenic signaling pathways. Moreover, EGCG enhances the treatment response of breast cancer cells to chemotherapeutic agents, indicating its potential as a complementary or adjuvant candidate for breast cancer management [20].

In summary, progress in understanding the molecular mechanisms governing breast cancer progression and tumor treatment responses has led to the discovery of new molecular targets and therapeutic approaches. The articles in this Special Issue explore various research areas related to these advancements, from identifying potential therapeutic targets to developing novel treatment strategies. The results presented in this Issue enhance our comprehension of the intricate molecular mechanisms involved in breast cancer and underscore the significance of unraveling these mechanisms.
